# Electronic Structure and Surface Properties of Copper Thiocyanate: A Promising Hole Transport Material for Organic Photovoltaic Cells

**DOI:** 10.3390/ma13245765

**Published:** 2020-12-17

**Authors:** Bonaventure A. Odeke, Gyang D. Chung, Jesutofunmi A. Fajemisin, Kabir S. Suraj, Denis K. Tonui, Ayinla R. Tobi, Thomas C. Bewaale, Jamiu A. Ajibola, Nelson Y. Dzade

**Affiliations:** 1 Department of Theoretical and Applied Physics, African University of Science and Technology, Km 10 Airport Road, Galadimawa, Abuja F.C.T. 900107, Nigeria; bodeke@aust.edu.ng (B.A.O.); gchung@aust.edu.ng (G.D.C.); jfajemisin@aust.edu.ng (J.A.F.); ksuraj@aust.edu.ng (K.S.S.); ktonui@aust.edu.ng (D.K.T.); ajamiu@aust.edu.ng (J.A.A.); 2 Department of Nanoscience and Nanotechnology, University of Grenoble Alpes, CS 40700 Grenoble, France; rtayinla@student.lautech.edu.ng (A.R.T.); tbewaale@aust.edu.ng (T.C.B.); 3 Department of Material Science and Engineering, African University of Science and Technology, Km 10 Airport Road, Galadimawa, Abuja F.C.T. 900107, Nigeria; 4 School of Chemistry, Cardiff University, Main Building, Cardiff CF10 3AT, UK

**Keywords:** copper thiocyanate (CuSCN), hole transport layer (HTL), dimethyl sulfoxide (DMSO), electronic structure, XPS valence band spectra, work function, density functional theory (DFT)

## Abstract

Considering the significance of hexagonal copper thiocyanate (β-CuSCN) in several optoelectronic technologies and applications, it is essential to investigate its electronic structure and surface properties. Herein, we have employed density functional theory (DFT) calculations to characterise the band structure, density of states, and the energy-dependent X-ray photoelectron (XPS) valence band spectra at variable excitation energies of β-CuSCN. The surface properties in the absence and presence of dimethyl sulfoxide (DMSO), a solvent additive for improving perovskite solar cells’ power conversion efficiency, have also been systematically characterised. β-CuSCN is shown to be an indirect band gap material (*E*_g_ = 3.68 eV) with the valence band edge demonstrated to change from being dominated by Cu-3*d* at soft X-ray ionisation photon energies to Cu-3*p* at hard X-ray ionisation photon energies. The adsorption energy of dimethyl sulfoxide (DMSO) on the (100) and (110) β-CuSCN surfaces is calculated at −1.12 and −0.91 eV, respectively. The presence of DMSO on the surface is shown to have a stabilisation effect, lowering the surface energy and tuning the work function of the β-CuSCN surfaces, which is desirable for organic solar cells to achieve high power conversion efficiencies.

## 1. Introduction

Halide perovskite solar cells (PSCs) are attractive, cost-effective, and earth-abundant candidates for solar cell development [1,2,3]. Because of the distinctive physical and optoelectronic properties, the organic–inorganic halide perovskites with typical ABX_3_ formula have attained certified overall conversion efficiencies exceeding 22% [4,5,6], making these materials suitable candidates to replace commonly employed Si-based solar cells. Perovskite solar cell device architecture consists of three important layers: the active photoabsorber (halide perovskite) layer, a hole-transporting layer (HTL), and an electron-transporting layer (ETL), to extract holes and electrons, respectively.

The hole-transporting layer helps to achieve efficient separation and transport of electrons and holes, which is essential for achieving high-performing perovskite solar cells. A large internal surface area of the active *n*-type photoabsorber layer can be used when pores in it can be filled entirely with a *p*-type HTL layer [7]. Although Spiro-OMeTAD is one of the most widely utilised HTL materials in PSCs [8], its instability limits peak performance attainment [9,10,11,12,13]. Tailored poly(3,4-ethylenedioxythiophene) polystyrene sulfonate (PEDOT:PSS) is another commonly used HTL material for high-efficiency perovskite solar cells [14,15,16,17]. Despite its many desirable properties, the sensitivity of the surface properties, including the work function, to processing conditions remain major challenges of PEDOT: PSS [18,19,20].

Copper(I) thiocyanate (CuSCN) is one of the promising hole-transporting *p*-type semiconductors owing to its chemical stability, nontoxicity, low-cost, high visible light transparency, and high hole conductivity (≥5 × 10^−4^ Scm^−1^) [21,22,23,24]. CuSCN can also be easily synthesised at high purities and it can be chemically modified because of its quasimolecular nature [25,26]. Due to its ability to effectively fill the pores/cracks in active *n*-type absorber layers, CuSCN can promote efficient transport of photogenerated carriers [7,27,28,29,30]. By using CuSCN as the hole extraction layer, Arora et al. achieved not only excellent thermal stability but also high stabilised efficiency exceeding 20% [21]. CuSCN has also attracted a surge of interest in light-emitting diodes (LEDs) [31,32], photodetectors [33,34], and field-effect transistors (FETs) [35].

Owing to the relevance of CuSCN to several critical technological applications, the fundamental electronic structure and surface properties must be comprehensively characterised and understood. This work presents a density functional theory (DFT) characterisation of the electronic structures: band structure, density of states (DOS), and X-ray photoelectron (XPS) valence band spectra at variable excitation energies of β-CuSCN. The simulated valence band spectra are compared with photoelectron spectroscopy measurements, which provide information on the electronic structure. Although DFT calculated density of states (DOS) have been used to interpret experimentally measured valence photoelectron spectra of β-CuSCN [36], there is a clear limitation as the orbital energies obtained from first principles DFT calculations are not excitation energies. There is, therefore, the need to determine the valence band spectra of β-CuSCN at variable excitation energies that are comparable to those of conventional X-ray sources (e.g., Al Kα) and hard X-ray photoemission spectroscopy, which are more bulk-sensitive. The valence band edge of β-CuSCN is indeed shown in the present study to change from being dominated by Cu-3*d* at soft X-ray ionisation photon energies to Cu-3*p* at hard X-ray ionisation photon energies. The surface properties of β-CuSCN with and without dimethyl sulfoxide (DMSO) modification are characterised by determining the most stable adsorption geometries of DMSO on the most stable (100) and (110) β-CuSCN surfaces and investigating the adsorption effects on tuning the surface work function. A wide range of work function tuning is essential for obtaining a good energy level alignment between the β-CuSCN HTL and the absorber perovskite material, and hence improved device characteristics. 

## 2. Materials and Methods 

The DFT calculations were performed in the VASP—Vienna Ab initio Simulation Package [37,38,39]. The interactions between the valence and core electrons are described using the projected augmented wave (PAW) method [40]. The Perdew–Burke–Ernzerhof (PBE) exchange-correlation function is used for geometry optimisations [41]. To accurately predict the electronic band gap and density of states (DOS) features of CuSCN, the screened hybrid functional HSE06 [42] with an exchange value of 25% was used. A kinetic energy cut-off of 600 eV and Monkhorst-Pack [43] K-points mesh of 7 × 5 × 3 converge the total energy of β-CuSCN to within 10^−6^ eV, and the residual forces on all relaxed atoms reached 10^−3^ eVÅ^−1^.

The METADISE code [44] was used to create the (100) and (110) β-CuSCN surfaces. The stability of the surfaces is quantified by calculating the surface energy (γ_r_) of the relaxed naked surfaces as:

(1) $$\gamma_{r}=\frac{E_{surf}^{relaxed}-nE_{bulk}}{2A}$$

where $E_{surf}^{relaxed\;}\;$ is the total energy of the relaxed surface, $nE_{bulk}$ is the energy of an equal number (*n*) of the bulk β-CuSCN atoms, and *A* is the surface area. To avoid interactions between periodic slabs, a 20 Å vacuum is added to the c-direction. To ascertain whether the adsorption of DMSO has a stabilisation effect on the surface, we have also computed surface energy of the one-side (1 × A) DMSO-covered β-CuSCN surface, calculated as:

(2) $$\gamma_{DMSO}=\frac{E_{surf+DMSO}^{relaxed}-E_{DMSO}-nE_{bulk}}{A}-\frac{E_{surf}^{relaxed}-nE_{bulk}}{2A}$$

where $E_{surf+DMSO}^{relaxed}\;$ is the total energy of the DMSO-covered surface and $\;E_{DMSO}\;$ is the total energy of the free DMSO molecule. The adsorption energy (*E*_ads_) of DMSO on β-CuSCN surfaces is calculated as:

(3) $$E_{ads}=E_{surf+DMSO}^{relaxed}-\left(E_{surf}^{relaxed}+E_{DMSO}\right)$$

Negative or positive adsorption energy denotes an exothermic or endothermic adsorption process, respectively. The averaged electrostatic potential along the c-direction of the (100) and (110) β-CuSCN surfaces was determined using the MacroDensity package [45,46]. The work function (Φ), the minimum energy required to eject an electron from a solid into vacuum, is determined as: Φ = E_V_ − E_F_, where E_v_ is the vacuum level and E_F_ is the Fermi level. Dipole corrections were accounted for in all surface calculations to ensure accurate determination of the vacuum level’s potential [47]. The XPS valence band spectra were simulated using the GALORE code [48], which used the Gelius model [49,50] to apply weightings to the atomic projected density of states (PDOS) based on the photoionisation cross-sections formulated by Scofield [51,52].

## 3. Results and Discussion

### 3.1. Crystal Structure β-CuSCN

**Figure 1:**
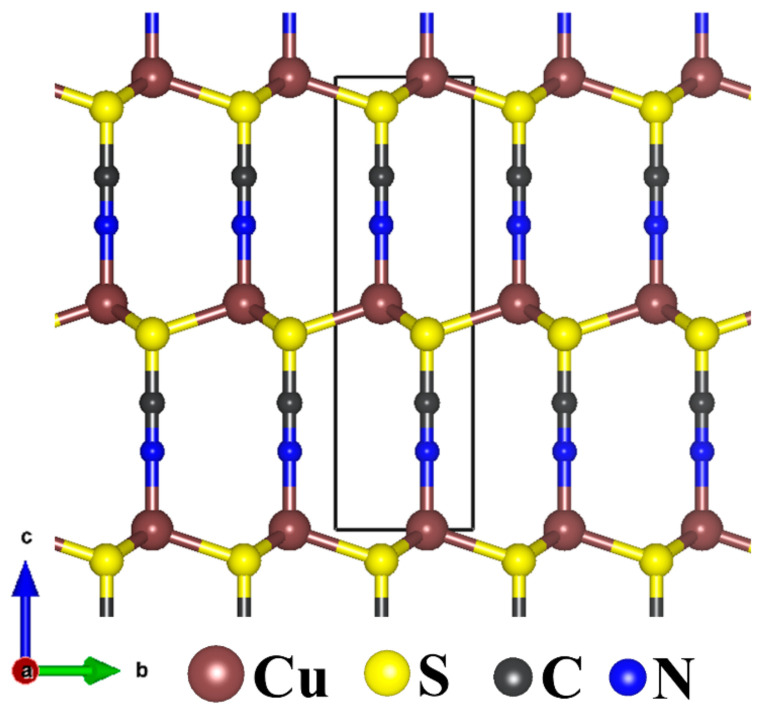
The hexagonal crystal structure of β-CuSCN.

CuSCN crystallises in two polymorphic forms, α-CuSCN (orthorhombic crystal lattice) and the β-CuSCN (hexagonal crystal structure) [53]. In the more stable β-CuSCN structure (Figure 1), the plains of Cu atoms are separated by layers of SCN ions, three-dimensionally interconnected by strong Cu–S bonds. As shown in Table 1, the lattice parameters of β-CuSCN are predicted at *a* = *b* = 3.828 Å and *c* = 10.970 Å, in close agreement with experimental data (*a* = *b* = 3.850 Å and *c* = 10.938 Å) [51] and previous DFT calculation results [36,54,55]. The average Cu–N, Cu–S, C–S, and C–N bond distances are calculated at 1.887, 2.316, 1.175, and 1.660 Å, respectively. The α(CuSC) and α(CuSCu) bond angles are predicted at 109.2° and 109.7°, respectively. 

### 3.2. Electronic Properties of β-CuSCN

**Figure 2:**
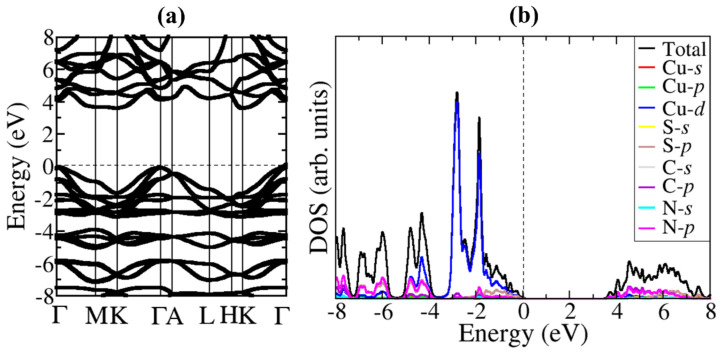
(**a**) Electronic band structure and (**b**) projected electronic density of states (PDOS) of hexagonal β-CuSCN.

β-CuSCN has an indirect band gap of 3.68 eV, as evident in the band structure (Figure 2a). The hybrid HSE06 functional predicted band gap compares closely with commonly reported experimental values in the range of 3.6–3.9 eV [56,57,58,59] and previous theoretical calculations [36,54,55]. The partial density of states (PDOS) of β-CuSCN is shown in Figure 2b. The valence band maximum (VBM) is dominated by Cu-3*d* and S-2*p* states, whereas conduction band minimum (CBM) is composed mainly of C-2*p* and N-2*p*, with a small contribution from C-3*d*. To gain insight into the superior hole-transport property of β-CuSCN, we have computed the effective masses of holes ($m_{h}^{*}$) and electrons ($m_{e}^{*}$) by fitting a quadratic polynomial to the energy versus reciprocal lattice vector *k*: $m_{e\left(h\right)}^{*}=\pm \;\hbar^{2}{\left(\frac{d^{2}E_{k}}{dk^{2}}\right)}^{-1}$. As shown in Table 2, the predicted $m_{h}^{*}$ (at the valence band maximum) and $m_{e}^{*}$ (at the conduction band minimum) in the Brillouin zone’s selected directions are generally very small, which point to their superior transport. The smallest effective masses of holes ($m_{h}^{*}$) appear in the direction from Γ to A (0.0037), whereas the highest appears in the Γ−M (0.1756) direction. These suggest that photogenerated holes in β-CuSCN should be easiest to transport along the Γ−A direction and least mobile in the Γ−M direction. 

**Figure 3:**
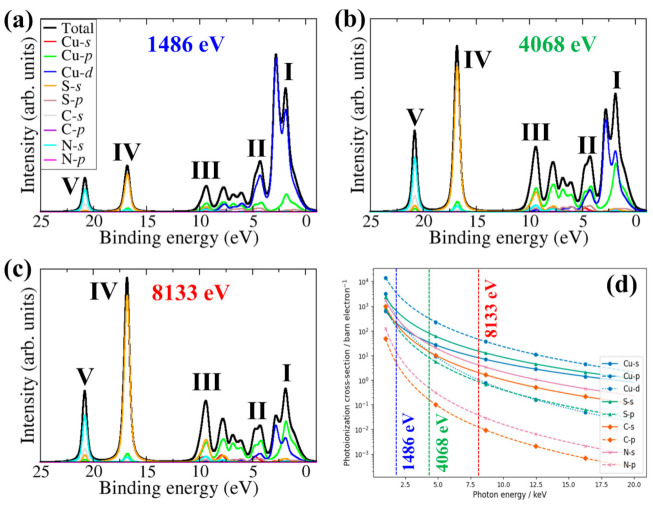
DFT-simulated XPS valence band spectra of hexagonal β-CuSCN obtained at (**a**) 1.486 keV (Al Kα_1_), (**b**) 4.068 keV, and (**c**) 8.133 keV ionizing photon energy. The dependence of the photoionisation cross-section on the ionising photon energy for the atomic orbitals is shown in (**d**).

The energy-dependent XPS valence band spectra of β-CuSCN at variable excitation energies (Al Kα1 (1486 eV), 4068 eV, and 8133 eV), obtained by applying weightings to the DFT-PDOS using the Scofield [51] formulated photoionisation cross-sections are shown in Figure 3. The chosen photon energies correspond to moving from soft (1486 eV) to intermediate (4068 eV) to hard (8133 eV) X-ray ionisation photon energy (hν). Five spectral features are assigned: I for the main valence band region (onset from 0.0 eV and extends to 3.4 eV), II, III, IV, and V for the broad satellite features located around 4, 9, 17, and 21 eV binding energies, respectively. The intensity of I relative to IV and V decreases continuously for increasing photon energy, which resulted in modifying the main valence band spectrum’s shape and increasing the broad satellite features’ relative intensity. The changes in the valence band spectral features may be attributed to the photon energy dependence of the photoionisation cross-section to the photon energy dependence of the photoionisation cross-section of the atomic *s*, *p*, and *d* orbitals that contributes to the valence band. Cu-3*d* states dominate the main valence band region (I) for β-CuSCN at lower photon energies, owing to their higher photoionisation cross-section (Figure 3d). The Cu-3*d* photoionisation cross-section decreases much more rapidly than Cu-3*p* with increasing photon energy, so its contribution becomes less significant (Figure 3a–c). This resulted in the main valence band (I and II) switching from being dominated by Cu-3*d* to Cu-3*p*. The satellite broad-feature IV can be assigned to the main contribution from S-3*s*, whereas the broad-feature V is assigned to contributions from N-2*s*. The simulated main valance band features at soft X-ray ionisation photon energy (1.486 keV) shows good agreement with the XPS valence band spectrum of CuSCN thin films reported by Jaffe et al. [36], which demonstrate that Cu-3*d* states dominate the main valence band region for β-CuSCN at lower photon energies. These results are also consistent with the work of Wijeyasinghe et al. [57], who showed that Cu-3*d* peaks dominate the main valence band. 

### 3.3. Surface Properties

**Figure 4:**
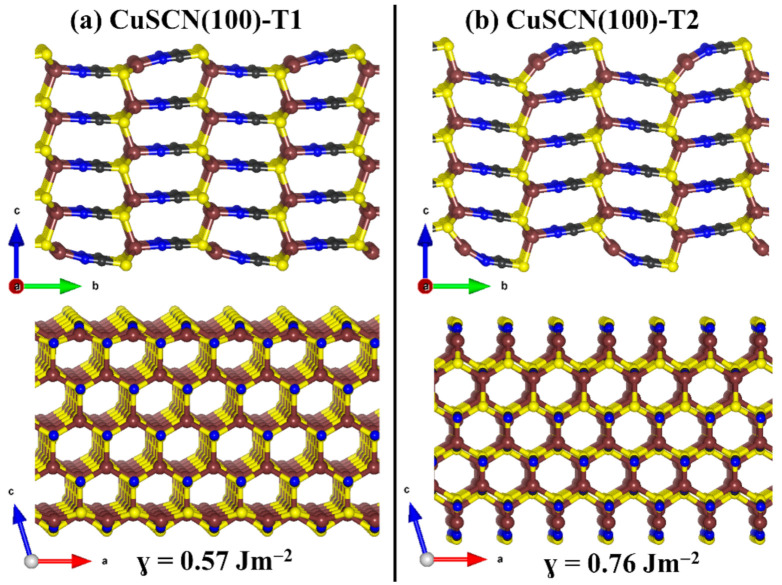
Side views of the optimised structures of the two non-dipolar (**a**) T1 and (**b**) T2 terminations of β-CuSCN (100) surface. Atomic colour: brown = Cu, grey = C, blue = N, and yellow = S.

**Figure 5:**
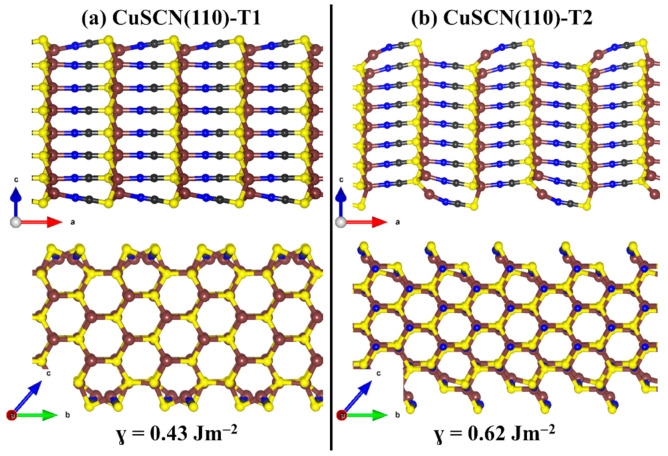
Side views of the optimised structures of the two non-dipolar (**a**) T1 and (**b**) T2 terminations of β-CuSCN (100) surface. Atomic colour: brown = Cu, grey = C, blue = N, and yellow = S.

Here, we have characterised the structure, composition, relative stabilities, and work function of the two most stable β-CuSCN (100) and (110) surfaces. Both surfaces have two non-dipolar terminations (denoted as termination T1 and T2), both of which are characterised to determine the most stable one. Shown in Figure 4 and Figure 5 are the relaxed structures of the two terminations of the (100) and (110) β-CuSCN surfaces. Termination T1 (T2) of the (100) surface has a calculated surface energy of 0.57 (0.76 Jm^−2^), indicating that the termination T1 is more stable than termination T2. Similarly, we predict the surface energy of T1 (T2) of the (110) surface to be 0.43 (0.62 Jm^−2^). Similarly, results were reported by Chen et al. [54], who reported surface energy of 0.74 and 0.63 Jm^−2^ for the most stable terminations of the (110) and (100) surfaces, respectively. 

**Figure 6:**
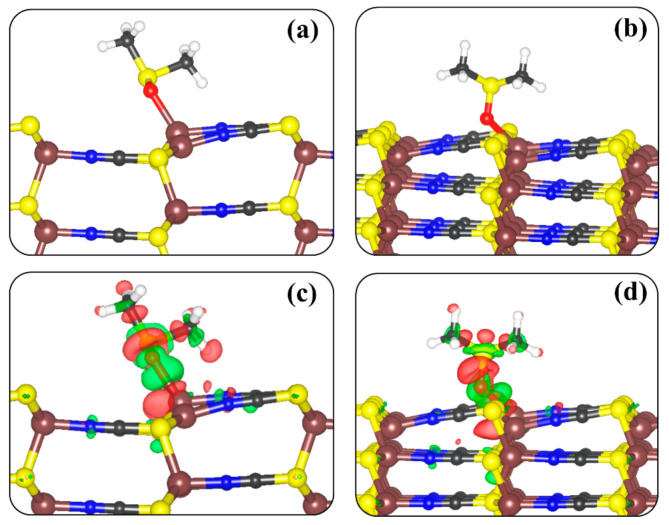
Lowest-energy DMSO adsorption geometry on (**a**) (100) and (**b**) (110) β-CuSCN surfaces. The corresponding differential charge density iso-surface contours are shown in (**c**) for (100) and (**d**) for (110) surface. Iso-surface contour colour: electron density accumulation (green) and depletion (red) by 0.02 e/Å^3^. Atomic colour: brown = Cu, grey = C, blue = N, and yellow = S.

Next, we investigated the adsorption characteristics of dimethyl sulfoxide (DMSO) on the most T1 termination of the β-CuSCN (100) and (110) surfaces to ascertain adsorption effects on the surface stability and work function. These insights are essential, considering that residues of DMSO solvent that is typically used to fabricate solution-processable halide perovskite materials [60] may adsorb onto the HTL β-CuSCN surfaces, altering the electronic properties. The DMSO molecule is introduced to the (100) and (110) surfaces in different orientations and at various sites to predict the most stable adsorption geometries, as shown in Figure 6. At both surfaces, the DMSO interacts with the Cu sites through the O atom, releasing an adsorption of −1.12 and −0.91 eV at the (100) and (110) surfaces, respectively. The interacting Cu–O bond distance at the (100) surface is calculated at 2.126 Å, whereas at the (110) surface, it is 2.191 Å (Table 3). Our predicted adsorption structures and energetics of DMSO at the β-CuSCN (100) and (110) surfaces are in good agreement with those reported by Zhang and Chen et al. [61], who show that the solvent DMSO molecule exhibits reasonable interactions with the CuSCN surfaces, predicting adsorption energy of 1.28 and 0.84 eV at the (001) and (110) surfaces, respectively.

The adsorption of the DMSO on the (100) and (110) surfaces is demonstrated to be characterised by redistribution of charge density in the surface-DMSO systems, as obtained from analysis of the differential charge density (∆ρ) iso-surface contours (Figure 6c,d). $∆\rho =\rho_{surf+DMSO}-\left(\rho_{surf}+\rho_{DMSO}\right)$, where $\rho_{surf+DMSO}$,$\;\rho_{surf}$, and $\rho_{DMSO}$ are the charge density of the total surf-DMSO complex, the naked β-CuSCN surface, and the free DMSO molecule as in the relaxed adsorbed configuration. We observe charge density accumulation between the Cu–O bonds, suggesting chemisorption. Bader charge analyses reveal that the net charge transfer from the β-CuSCN surfaces to the DMSO molecule is minimal, calculated to be 0.08 and 0.05 e^−^ at the (100) and (110) surfaces, respectively (Table 3). Owing to the small charge transfer to the DMSO molecule upon adsorption, the interatomic bond distances and angles changed marginally, relative to the gas-phase parameters.

**Figure 7:**
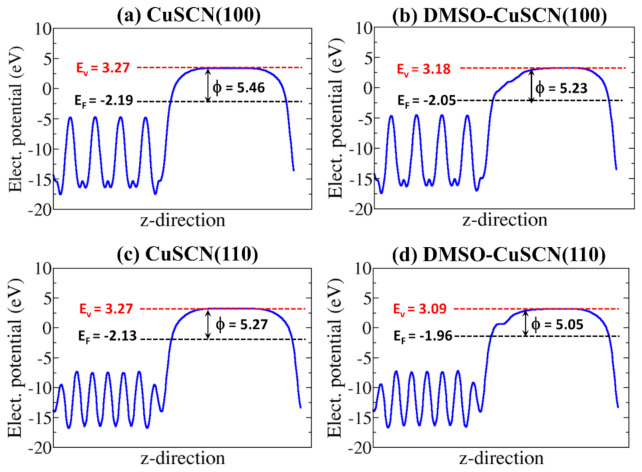
The averaged electrostatic potentials for (**a**) naked CuSCN (100), (**b**) DMSO-covered CuSCN (100), (**c**) naked CuSCN (110), and (**d**) DMSO-covered CuSCN (110) surfaces. Red dashed line = vacuum level (*E*_vac_), black dashed line = Fermi level (E_F_), and Φ = work function.

The DMSO-covered surfaces are predicted to have smaller surface energies compared to the naked surfaces, suggesting that the DMSO adsorption has a stabilisation effect on the (100) and (110) β-CuSCN surfaces. The DMSO-covered β-CuSCN(100) surface energy is calculated at 0.49 Jm^-2^, compared to 0.57 Jm^−2^ for the naked surface. Similarly, the DMSO-covered β-CuSCN (110) surface has a surface energy of 0.39 Jm^−2^, compared to 0.43 Jm^−2^ for the naked surface. The adsorption of DMSO on the (100) and (110) β-CuSCN surfaces modifies their work function (Φ). Hole transport materials with a high degree of tunable work functions that permit proper energy alignment with the donor materials’ deep valence bands are desirable for high-efficiency organic solar cells [62]. The Φ of the naked CuSCN (100) surface is computed at 5.46 eV vs. vacuum, as shown in Figure 7a, compared to 5.23 eV for the DMSO-covered (100) surface (Figure 7b). For the CuSCN (110) surface, the Φ naked and DMSO-covered surfaces are predicted at 5.27 and 5.05 eV, respectively (Figure 7c,d). 

The reduction in the work function upon DMSO adsorption may be rationalised by considering the fact that the DMSO adsorption is characterised by electron density rearrangement, which smoothens the surface electric charge distribution according to the Smoluchowski effect [63,64]. Similar results have been reported for other organic functionalised surfaces [65,66,67,68]. These calculated work functions compare closely with experimental measurements, which report the Φ of CuSCN in the range of 5.0–5.4 eV [69,70,71,72]. Wijesekara et al. [70] reported the work function of CuSCN thick film to be 4.82 eV. Dzikri et al. [71] and Kim et al. [72] reported the work functions of 5.53 and 5.4 eV, respectively. The predicted work function of CuSCN also compares with those of other commonly used hole transport layer materials: Spiro-OMeTAD = 4.9, PEDOT:PSS = 5.1 eV, NiO_x_ = 5.4 eV, V_2_O_5_ = 5.4 eV, MoO_x_ = 5.3 eV, WO_3_ = 5.35 eV, and GO = 4.9 eV [73,74]. The close match between the work function of CuSCN and the highest occupied molecular orbital level of most perovskite materials (e.g., CH_3_NH_3_PbI_3_ = –5.4 eV) ensures good alignment of energy levels, which will give rise to improvements in device efficiency. 

## 4. Summary and Conclusions 

In summary, we have comprehensively characterised the electronic structure, energy-dependent XPS valence band spectra at variable excitation energies, and the surface properties of β-CuSCN in the absence and presence of dimethyl sulfoxide (DMSO), using first principles density functional theory calculations. The β-CuSCN is shown to possess an indirect large band gap (E_g_ = 3.68 eV) with the valence band edge demonstrated to change from being dominated by Cu-3*d* at soft photon energies to Cu-3*p* at hard X-ray ionisation photon energies. The adsorption of DMSO on the (100) and (110) β-CuSCN surfaces was demonstrated to have a stabilisation effect, lowering the surface energy. The adsorption was shown to be characterised by electron redistribution in the DMSO-CuSCN systems, leading to accumulation within the interacting Cu–O bonding regions. The presence of the DMSO was shown to tune the work function of the CuSCN surfaces, which is desirable for hole transport layer materials to achieve improved solar device characteristics and performance. 

## Figures and Tables

**Table 1:** Calculated lattice parameters of β-CuSCN in comparison with experiment and previous density functional theory (DFT) data.

	Exp.	GGA + D3	GGA	GGA + D2	HSE06	GGA
Reference	[53]	This Work	[36]	[54]	[54]	[55]
a (Å)	3.850	3.828	3.781	3.832	3.960	3.857
c (Å)	10.938	10.970	10.987	11.023	11.055	10.979
c/a	2.841	2.865	2.906	2.876	2.791	2.847

GGA—Generalized Gradient Approximation.

**Table 2:** Predicted effective masses of holes (m*_h_) and electrons (m*_e_) of β-CuSCN along high symmetry directions.

Direction	m*_h_ (m_e_)	m*_e_ (m_e_)
Γ−M	0.1756	0.0066
M−K	0.0189	0.0379
K−Γ	0.0146	0.0049
Γ−A	0.0037	0.4921
A−L	0.0350	0.0049
L−H	0.0068	0.0793
H−K	0.0079	0.00131

**Table 3:** Calculated adsorption energies (*E*_ads_), charge transfer (∆q), and representative interatomic distances of the DSMO adsorption complex on β-CuSCN (100) and (110) surfaces.

Parameter	DMSO (Gas-Phase)	CuSCN (100)	CuSCN (110)
*E*_ads_ (eV)		−1.12	−0.91
∆q (e^−^)		0.08	0.04
*d*_Cu–O_ (Å)		2.126	2.191
*d*_S–O_ (Å)	1.520	1.525	1.519
*d*_S–C_ (Å)	1.789	1.807	1.808
α(OSC) (°)	121.7	104.2 (106.0)	104.7 (105.4)
α(CSC) (°)	116.6	98.7	98.3
